# Impact of Grinding and Sorting Particle Size on Phytochemical Yield in *Dipterocarpus alatus* Leaf Extract

**DOI:** 10.1155/2023/4512665

**Published:** 2023-12-22

**Authors:** Kritsadang Senawong, Somporn Katekaew, Suchat Juntahum, Kittipong Laloon

**Affiliations:** ^1^General Education Teaching Institute, Khon Kaen University, Khon Kaen 40002, Thailand; ^2^Department of Biochemistry, Faculty of Science, Khon Kaen University, Khon Kaen 40002, Thailand; ^3^Department of Agricultural Engineering, Faculty of Engineering, Khon Kaen University, Khon Kaen 40002, Thailand; ^4^Food, Energy, Water Security Research Institute, Khon Kaen University, Khon Kaen 40002, Thailand

## Abstract

The main objective of this study was to investigate the impact of grinding (pretreatment) with a pin mill on the crude extract yields of *Dipterocarpus alatus* (Yang-Na) leaves. A factorial design in a completely randomized design was conducted to study the combinational effects of sieve sizes (1.0, 1.5, and 3.0 mm) and feed rates (1.0, 1.5, and 3.0 kg min^−1^), examining the interaction of parameters for grinding oven-dried Yang-Na leaves. Ethanol extraction initially evaluated the influence of Yang-Na leaf powder with diverse particle sizes. When sorting particle size, the crude extract yield increased as the particle size decreased, with 0.038–0.150 mm particles yielding the highest extraction, although yields decline when the particle size is lower than 0.038 mm. The average particle sizes, production capacity, and fineness modulus all exhibited a significant decrease as the sieve size and feeding rate were reduced, while the specific energy consumption showed an inversely proportional relationship with these parameters. Intriguingly, the crude extract yield remained independent of the average particle size. Notably, the highest yield (14.79 g kg^−1^) was derived from a 0.31 mm average particle size, ground with a 1.5 mm sieve and a 3 kg min^−1^ feeding rate. This suggests that the pretreatment, involving both grinding conditions and sorting size, has an impact on the performance of the extraction process. However, this study offers an energy-efficient alternative, advocating for using average particle sizes without prior sorting, streamlining the extraction process while maintaining substantial yields. These insights underline the crucial influence of particle size and grinding techniques, advancing our understanding of efficient herbal extraction techniques for industrial applications.

## 1. Introduction

*Dipterocarpus alatus*, commonly known as the resin tree or Yang-Na, is native to various Southeast Asian countries [1], which is extensively utilized for ecological, economic, and medicinal purposes [2]. Yang-Na has a longstanding tradition of ethnomedicinal use. Its bark has been employed in the treatment of ailments such as rheumatism, liver diseases, and as an appetite stimulant for cattle [3]. This tree is oleoresin-rich wood, making it the extraction of resin, which serves as an alternative energy source for agricultural diesel engines [4]. Moreover, the medicinal potential of Yang-Na has been explored extensively, with its bioactive compounds showing promise in preventing and treating various conditions, including cancer, bacterial infections, human immunodeficiency virus (HIV), and inflammation [2, 5–10]. In addition, these bioactive compounds have found applications in cosmetics, serving as key components in perfume and makeup foundation products [1, 2]. The phytochemical composition of Yang-Na has been a subject of scientific interest, revealing a rich array of phenolic compounds, particularly sesquiterpenes, triterpenes, oligostilbenoid, coumarin derivatives, resveratrol, and phytosterol [11]. Notably, various studies have reported the presence of compounds such as *ɛ*-viniferine, *α*-viniferine, vaticanol B, and hopeaphenol in the bark of *D. alatus* [12, 13]. Furthermore, the bark resin of Yang-Na is rich in gurjun essential oils such as *α*-gurjunene and *β*-gurjunene, which have found utility in cosmetics for enhancing color saturation and gloss [14]. The presence of such phenolic compounds in Yang-Na holds promise for skin applications, particularly in reducing the impact of free radicals responsible for skin aging, diseases, and damage such as wounds and burns [15]. In addition, flavonoids identified in the leaves of Yang-Na have the potential to exert antioxidant and antineuroinflammatory effects, which could be relevant to depression pathophysiology [16, 17].

Biologically active natural compounds are molecules produced by plants or plant-related microbes, such as endophytes [18, 19]. In recent times, plant extracts have garnered significant attention for their potential medicinal applications due to their diverse phytochemical composition and associated health benefits. For Yang-Na, it can be known that the phytochemical compounds are from whole parts of tree. Several parts of Yang-Na, viz., leaves, twigs, and bark, were extracted with alcoholic to crude yield percentages of 11.80, 6.07, and 4.24, respectively [9]. This indicates that leaves provided the highest crude extract yield compared to other parts. Moreover, Yang-Na leaves can be harvested abundantly without damaging the tree, setting them apart from other plant parts. Therefore, the leaves of the plant were considered a potential part to use as the source of crude bioactive substances.

The extraction process is the first step to play a pivotal role in obtaining phytochemical compounds from various biomaterials. In the process of extraction, after oven-drying, the leaves must undergo coarse crushing before extracting bioactive compounds. At the laboratory level where extraction is made, it uses a small electric fine blender for grinding with little per time and without sorting particle sizes, resulting in low yield and productivity efficiency. Moreover, the efficiencies of extraction methods depend on some critical parameters from pretreatment to final extraction such as grinding, drying, extraction temperature, time, biomass percentage, material particle size, and solvent type. It is reported that the material particle size usually has a significant effect on the efficiency of the extraction process [20]. In the future, if the demand for bioactive compounds from Yang-Na increases, the production of the coarse dried grinding of Yang-Na leaves (CGY) must increase; there is a need to understand every aspect during the extraction process of the Yang-Na leaves, especially the particle size. The currently used small electric fine blenders in laboratories may not be suitable for large-scale production of CGY, necessitating the need for specialized equipment. Moreover, the effect of grinding to obtain a suitable particle size of CGY for extraction needs clarification. Although grinding equipment such as hammer mills, disc mills, roller mills, and pin mills are ably applied for grinding Yang-Na leaves, there is no equipment to demonstrate the effect on the preparation process of CGY. In addition, there is a need to clarify the effect of separation of each particle size to produce CGY that is suitable for extraction.

Because particle size is an important parameter to influence the phytochemical extraction system in terms of the amount, composition, and biological activity, preliminary plant grinding into fine particles can be carried out [21]. The reduction of particle size by milling is a crucial step to change the physical, chemical, functional, structural, and biological properties of raw material [22], enhancing the overall efficiency of the entire extraction process [23]. Amoura et al. [24] reported that wet milling resulted in increased oil binding capacity and emulsion stability, reduced water binding capacity and foam stability of sorghum grain flour compared to dry milling, and improved water- and oil-binding capacities of extracted kafirins by 17% and 5%, respectively. Therefore, pretreatment (fine grinding and sieving) should be considered before extraction, as it leads to a differential distribution of bioactive compounds based on particle size [25]. For Yang-Na, grinding was conducted by using a small electric fine blender without sorting. Subsequently, it was macerated with alcohol, filtered with filter paper, and evaporated to obtain the crude extract. [9, 26]. In this condition, the effect of particle size or energy consumption on the grinding process is not investigated.

This research seeks to shed light on the importance of particle size manipulation in the extraction process of Yang-Na leaves, with potential implications for enhancing the efficiency and yield of bioactive compounds from this valuable medicinal plant. To maximize the utilization of bioactive compounds from Yang-Na, there is a need to understand every aspect of the extraction process, especially the grinding process for productively enhancing the performance of the extraction method. Moreover, an optimal particle size of CGY for extraction has not been reported yet. Therefore, the objective of this study was to determine the impact of particle size on the extraction yield of Yang-Na leaves. Subsequently, the effect of milling factors was investigated for grinding Yang-Na leaves before the extraction steps, aiming to facilitate large-scale production of CGY.

## 2. Materials and Methods

The research methodology of this study was separated into 3 parts: (1) preparation of dried Yang-Na leaves, (2) characterization of CGY, and (3) designing and developing the process of grinding dried Yang-Na leaves.

### 2.1. Preparation of Dried Yang-Na Leaves

Mature and healthy Yang-Na leaves, characterized by an ovate-lanceolate shape, 6–15 cm in width, 12–35 cm in length, 11–18 (−20) pairs of secondary veins, and green-black petioles, were collected from trees aged between 3 and 20 years. The collection site was situated at Khon Kaen University in Khon Kaen, Thailand (16°27′34.3″N 102°48′56.9″E, 200 m above mean sea level). After that, Yang-Na leaves were washed with tap water 2-3 times and then left in an indoor area to allow water to drain off. Subsequently, the leaves were oven-dried at 70°C for 72 hours or until the dry biomass reached a constant weight. Subsequently, the dried Yang-Na leaves were stored in black plastic bags at room temperature for further experiments.

### 2.2. Characterization of CGY

The dried Yang-Na leaves were ground using an electric herb miller (COSUAI CS-700, China). The resulting CGY was sieved through a series of sieves with aperture size numbers of No. 4 (4.75 mm), No. 8 (2.360 mm), No. 16 (1.180 mm), No. 30 (0.600 mm), No. 50 (0.300 mm), No. 100 (0.150 mm), No. 200 (0.075 mm), and No. 400 (0.038 mm), along with a pan, respectively, and then stacked according to the American Society for Testing and Materials (ASTM) C136 standard method. The sample was prepared immediately before bioactive extraction.

### 2.3. Determination of Crude Extraction Yield

The crude extraction yield of Yang-Na of different particle sizes from >1.18 to >0.075 mm was determined by the ethanoic method. Specifically, 100 g of CGY was mixed with 400 mL of 99.5% ethanol and shaken at 150 rpm and 20°C for 24 hours. The suspension was then poured into a Buchner funnel with Whatman filter paper #1. Afterward, the suspension was evaporated with a rotary evaporator at 45°C and 50 rpm until the remaining volume reached about 10% of the initial volume. The extract was further dried in an oven at 60°C for 24 hours. The crude extraction yield was weighed by deducting the container weight, and the obtained extract pastes were stored at 4°C until further use.

### 2.4. Determination of Factors for Grinding Yang-Na Leaves

**Figure 1:**
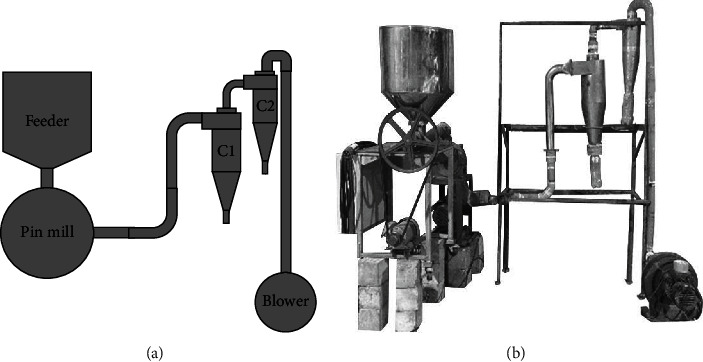
Diagrammatic (a) and machines (b) for grinding Yang-Na leaves.

The dried Yang-Na leaves were initially subjected in a hammer mill with 12 beating blades, 3 mm of sieve size, 1 horsepower of the motor, 1430 rpm, and a feed rate of 6 kg hr^−1^ for reduction of the leaf size. The factors affecting ground Yang-Na leaves were conducted by using a pin mill with the standard production capacity (5 kg min^−1^) and rotational speed (5,500 rpm). The feeder machine was assembled on a pin mill to control and adjust the feed rate. Cyclone separators are connected from pin mills to minimize air pollution. Cyclone separators were designed with a floating velocity of CGY of 0.3 mm which was 0.336 m s^−1^. It consists of a primary cyclone (C1) with a diameter of 20 cm and a secondary cyclone (C2) with a diameter of 15 cm. The particle size from C1 to C2 was uniformly collected to subject a sieve shaker according to the ASTM C136 standard method to calculate average particle sizes. The production line (machines) for grinding Yang-Na leaves is shown in Figure 1.

A factorial experiment was conducted by a completely randomized design (CRD) to study the combinational effect of sieve sizes and feed rates for grinding Yang-Na leaves. The treatments were the combination of 3 levels of sieve size (1.0, 1.5, and 3.0 mm) and 5 levels of the feeding rate (1, 2, 3, 4, and 5 kg min^−1^). The parameters evaluated included production capacity, specific energy consumption (SEC) of the size reduction process, average particle sizes, fineness modulus, and crude extraction yield:

(1) $$\begin{array}{c} \text{production capacity }\left(\%\right)=\frac{\text{weight of leaf powder after grinding }\left(\text{kg}\right)}{\text{weight of dried leaves }\left(\text{kg}\right)}\;\times 100,\\ \text{SEC }\left({\text{kWh kg}}^{-1}\right)=\frac{\text{total energy consumption }\left(\text{kWh}\right)}{\text{weight of leaf powder after grinding }\left(\text{kg}\right)}. \end{array}$$

The energy value of grinding was calculated at 5 THB per power unit (1 THB = 0.029 USD).

Average particle sizes were conducted according to the ASTM C136 standard method. In brief, 300 g of ground dried leaves was poured through a series of sieves with the following sieve numbers: No. 4, 8, 16, 30, 50, 100, 200, and 400, and a pan, respectively. Particle sizes were separated at 150 rpm for 10 min using a sieve shaker. The CGY on each of the sieves was weighed and recorded. The average particle sizes were then calculated using the following formula:

(2) $$\begin{array}{c} d_{\text{gw}}=\log^{-1}\left[\frac{\sum \left[w_{i}\;\log\;d_{i}\right]}{\sum w_{i}}\right], \end{array}$$

where *d*_gw_ = average of the particle size (mm), *d* = diameter of the sieve pore, and *w* = weight of the retained particle on each sieve

The percentage retained on each sieve was simply calculated by dividing the weight retained on each sieve by the total dry weight. Fineness modulus was calculated by adding accumulative % retained on each sieve and dividing the sum by 100 with the following formula:

(3) $$\begin{array}{c} \text{fineness modulus}=\frac{\sum \left(\text{accumulative}\;\%\;\text{retained on each sieves}\right)\;}{100}, \end{array}$$

where each sieve = sieve No. 4, 8, 16, 30, 50, 100, 200, and 400.

The crude extraction yield was investigated from obtained CGY in each treatment without sorting particle size by the method described in the characterization of the CGY topic. The crude extract value was calculated at 30,000 THB kg^−1^ (1 THB = 0.029 USD).

### 2.5. Statistical Analysis

All data, including production capacity, SEC, average particle sizes, fineness modulus, and crude extraction yield, were collected in triplicates and analyzed using factorial in CRD with the analysis of variance (ANOVA). Subsequently, it was performed using Fisher's least significant difference (LSD) test at *P* ≤ 0.05, using the Statistix 8.0 program.

## 3. Results and Discussion

### 3.1. Results

#### 3.1.1. Characteristic of CGY

**Figure 2:**
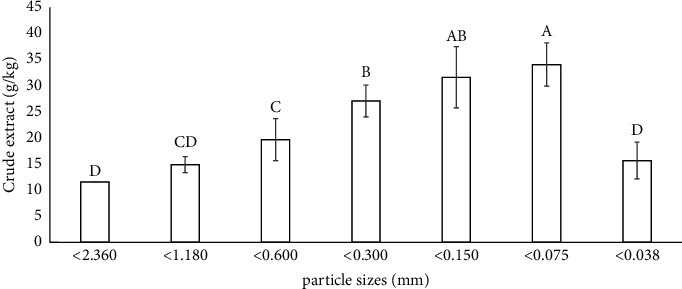
The effect of the particle size of CGY on crude extract yield. Different uppercase letters above the bars indicate a statistically significant difference at *P*≤0.05.

After grinding with a small electric fine blender, the Yang-Na leaf sizes were reduced to >0.038 to <2.360 mm. Overall, the crude extract yield of CGY showed a consistent increase as the particle size decreased. Notably, the highest crude extract yield of CGY (3.41%) was recorded at a particle size of 0.038–0.075 mm. However, the crude extract yield decreased for particles larger than this range. Surprisingly, the smallest particle size (0–0.038 mm) did not yield the highest extract yield (Figure 2). These findings indicate that particle sizes ranging from 0.038 to 0.150 mm are crucial for optimizing crude extract yield, while the particle sizes of 0.300–0.600 mm should also be considered for extraction.

#### 3.1.2. The Effect of Factors on Grinding Yang-Na Leaves

Table 1 shows the effect of grinding Yang-Na leaves with a hammer mill, followed by continuous grinding with a pin mill, on the percentage distribution of each particle size. After initial grinding with a hammer mill, the Yang-Na leaves were reduced in size from >0.038 to <2.360 mm, with most particles falling in the 1.18–2.36 mm range (46.22%). After secondary grinding with a pin mill, the coarse powder-dried Yang-Na leaves were ground to >0.038–2.36 mm, eliminating particles larger than 2.36 mm. In addition, using sieves of 1–1.5 mm in size led to a complete absence of particles >1.18 mm. Overall, as the sieve size and the feeding rate decreased, the obtained particle sizes of Yang-Na leaves reduced. For instance, grinding with 1 mm sieves and feeding rates of 1 and 2 kg min^−1^ resulted in the highest amounts of particles in the 0.3–0.6 mm range. While grinding with 1.5 mm sieves at a feeding rate of 1 kg min^−1^, the highest amounts of particle size were found at 0.3–0.6 mm. However, when using feeding rates of 2 and 3 kg min^−1^, the highest amount of particle sizes shifted to the range of 0.6–1.18 mm.

**Figure 3:**
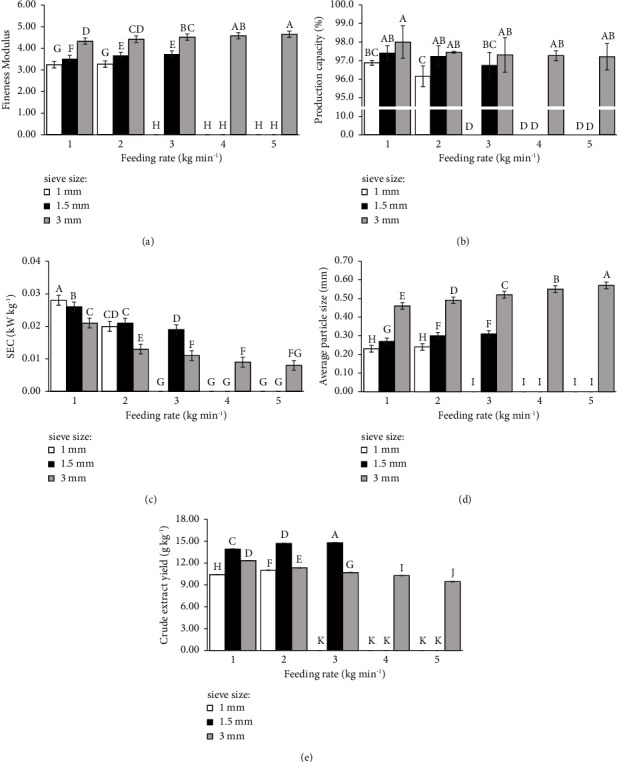
Effect of the feeding rate and sieve size using a pin mill on fineness modulus (a), production capacity (b), SEC (c), average particle size (d), and crude extract yield (e). Different uppercase letters above the bar indicate significant differences (*P* ≤ 0.05) among the data in each subfigure.

Notably, the machine encountered issues when grinding with a 1 mm sieve at feeding rates of 3, 4, and 5 kg min^−1^, as well as with a 1.5 mm sieve at feeding rates of 4 and 5 kg min^−1^. A particle size of 0.6 to 1.18 mm was obtained as the highest regardless of the feeding rates (1, 2, 3, 4, and 5 kg min^−1^) during grinding with 3 mm sieves. Based on the collected average particle size (Figure 3), employing the smallest sieve size and lowest feeding rate is recommended for grinding to obtain the smallest average particle size. However, other parameters should also be considered, especially the crude extract yield.

Figure 3 shows the effect of grinding the CGY with a pin mill on various parameters, including fineness modulus, production capacity, power, average particle size, and crude extraction yield and its value. Fineness modulus was significantly decreased in a 1 mm sieve size treatment and increased with larger sieve sizes and feeding rates. Production capacity increased with increasing sieve size, but when keeping the sieve size constant, it decreased with higher feeding rates. SEC decreased with increasing sieve sizes and feeding rates, and the highest SEC was recorded when using 1 mm sieves and 1 kg min^−1^ feeding rates. The average particle size significantly increased with increasing sieve sizes and feeding rates. Notably, the smallest average particle size (0.23 mm) was observed during grinding with 1 mm sieves and 1 kg min^−1^ feeding rates. The crude extract yield depended on the average particle size. For instance, grinding with 1 mm and 1.5 mm sieves led to an increase in the crude extract yield with increasing feeding rates, while 3 mm sieves showed a significant decrease in the crude extract yield with higher feeding rates. The highest crude extract yield (14.79 g kg^−1^) was noted in grinding with a 1.5 mm sieve and a 3 kg min^−1^ feeding rate, indicating that smaller particle sizes do not always yield higher extract yields.

The results showed that both sieve sizes and feeding rates, along with their interaction, significantly affected all parameters, including production capacity, SEC, particle sizes, fineness modulus, and crude extract yield (Table 2).

#### 3.1.3. Relationship between the Energy Value of Grinding and the Crude Extract Value

**Figure 4:**
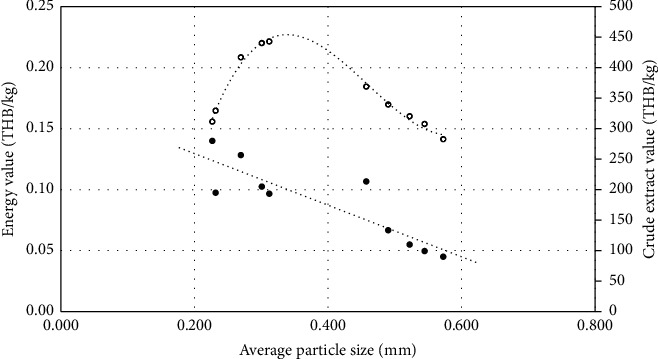
The relationships between the energy value of grinding (white circles) and the crude extract value (black circles) and average particle sizes.

Figure 4 shows the relationship between the energy value of grinding and the crude extract value in conjunction with average particle sizes. The results indicate that an average particle size of 0.31 mm is associated with the highest crude extract value and middle energy value for grinding, suggesting an optimal particle size for efficient extraction.

## 4. Discussion

### 4.1. Effect of Particle Size on Crude Extract Yields

This study presents a novel exploration into the impact of particle sizes of the oven-dried Yang-Na leaf powder on crude extract yields. The results revealed a clear relationship between the particle size and extract yield that showed smaller particle sizes produced higher extract yields (Figure 2). The remarkable impact of particle size on the accessibility of bioactive compounds was evident, with a notable increase observed as particle size decreased. In previous research on green tea leaves and macroalgae, smaller particle sizes demonstrated higher extract yields [20, 27, 28]. The reduction in particle size amplifies the contact surface area between the sample and the ethanol solvent used for extraction [29], thereby enhancing the extract yield. Moreover, the extracts from samples with smaller particle sizes produced significantly higher phytochemical content such as total phenolic and total flavonoid content [20, 30]. Nonetheless, it is worth noting that while reducing particle size generally enhances bioactive compound recovery, it may not always guarantee the highest yield. Smaller particle sizes in green tea powders led to an initial increase, followed by a reduction in accessible polyphenols and catechins, with the 75–180 µm size showing the optimal levels [31, 32]. As observed in this study, the smallest particle sizes (>0.038 mm) did not yield the highest crude extract yield (Figure 2). Possible explanations for this phenomenon include heat generation during superfine grinding, resulting in polyphenol and catechin oxidation as observed by Hu et al. [33], and the potential for powder agglomeration that hinders dissolution.

### 4.2. Effect of Grinding on Crude Extract Yields

This study underscores the critical role of sample pretreatment (grinding), specifically grinding, in influencing crude extract yields from oven-dried Yang-Na leaves. While a hammer mill is traditionally employed to reduce large particles to smaller sizes, this investigation focused on subsequent grinding using a pin mill, designed for processing hard materials with low moisture content. It is noteworthy that the effect of grinding with the hammer mill on particle size was not explored, considering its primary purpose of particle size reduction. After the preliminary reduction with a hammer mill, subsequent grinding with a pin mill was characterized by varying factors, viz., sieve sizes and feeding rates. The data in Figure 3 revealed that, generally, the average particle sizes, production capacity, and fineness modulus decreased as the sieve size and the feeding rate decreased, with specific energy consumption exhibiting an inverse relationship (Figure 3). However, it is worth mentioning that some machine operations in Figure 3 were hampered due to interactions between sieve sizes and feeding rates that surpassed the machine's grinding capability. An applied pin mill machine for grinding Yang-Na leaves showed a maximum capacity production of 5 kg min^−1^, highlighting its efficiency in terms of time and energy consumption compared to an electric fine blender typically used in laboratory settings. While successfully grinding, the pin mill production capacity averaged 96% that showed a minor fraction of samples was retained in the machine. The optimized conditions for grinding with the pin mill were using a sieve size of 1.5 mm and a feeding rate of 3 kg kg^−1^, challenging the assumption that the smallest average particle size necessarily corresponds to the highest yield, which was noted in sieve size 1 mm and feeding rate 1 kg min^−1^. The possible reason for this case was the complex, agglomeration, and absorption of particles during the ethanol suspension separation step of extraction, affecting the volume of the supernatant. Although the concentration of the crude extract per volume could be highest in the case of the smallest particles (data not shown), the total yield calculations presented in Figure 3 showed a different outcome. A previous study on the superfine ground apple powder revealed that agglomeration became more pronounced with prolonged grinding time, resulting in a gradual increase in particle size. This phenomenon could be attributed to the particle size of the powder reaching a specific threshold, causing an elevation in mutual surface adsorption forces, thereby facilitating intermolecular aggregation [34]. In this study, particle size sorting after grinding prior to extraction was avoided due to the requirement for effective sorting machines such as cyclone separators, vibrating filter screening machines, or other equipment, which would introduce additional process preparation steps and increase energy requirements. Consequently, the study opted for direct extraction using the average particle size, a strategy that streamlines the workflow, conserves energy, and maintains yields without causing a significant reduction in overall yields.

## 5. Conclusion

This study sheds light on the crucial influence of particle size and grinding techniques (various sieve sizes and feeding rates) on crude extract yields from oven-dried Yang-Na leaves. The crude extract was obtained through maceration with ethanol, followed by a filtration process and an evaporation process. The results concluded the following:

- The impact of sorting by particle size reveals that smaller particle sizes tend to result in higher yields; however, yields decline when the particle size is below 0.038 mm.
- Fineness modulus and average particle sizes decreased with a decrease in the sieve size and feeding rate, in contrast to SEC
- Production capacity increased with an increase in the sieve size and a decrease in the feeding rate
- The smallest average particle size collected from a sieve size of 1 kg and a feeding rate of 1 kg did not always yield a higher crude extract than larger particle sizes
- The crude extract yield significantly increased when using a sieve size of 1.5 mm and feeding rate of 3 kg, which is an optimal grinding condition
- The optimal grinding CGY without sorting demonstrated a particle size of 0.31 mm, providing the highest crude extract value while maintaining a low energy cost of grinding
- The findings for grinding medicinal plants present an energy-efficient approach, reducing sorting steps to maximize yield without sacrificing quality

## Figures and Tables

**Table 1:** Effect of size reduction with a hammer mill and continuous grinding with a pin mill on the percentage of each particle size.

Treatments		Percentage of particles through different sieve sizes							
Sieve size (mm)	Feeding rate (kg min^−1^)	2.360 (mm)	1.180 (mm)	0.600 (mm)	0.300 (mm)	0.150 (mm)	0.075 (mm)	0.038 (mm)	Pan
*Size reduction with a hammer mill*									
3	6	24.67	46.22	14.55	6.22	2.89	2.00	1.67	1.78

*Grinding with a pin mill*									
1.0 mm	1	0	0	11.77	47.43	17.23	9.57	6.57	7.43
	2	0	0	15.90	42.77	16.90	10.57	7.10	6.77
	3	—	—	—	—	—	—	—	—
	4	—	—	—	—	—	—	—	—
	5	—	—	—	—	—	—	—	—
1.5 mm	1	0	0	25.10	39.33	15.47	9.23	5.77	5.10
	2	0	0	37.00	31.77	11.90	8.00	5.23	6.10
	3	0	0	38.33	31.33	12.57	7.77	5.10	4.90
	4	—	—	—	—	—	—	—	—
	5	—	—	—	—	—	—	—	—
3.0 mm	1	0	10.67	47.00	24.13	8.77	5.1	2.56	1.78
	2	0	17.10	44.33	20.90	7.77	5.23	2.44	2.22
	3	0	23.10	40.33	19.23	7.90	5.1	2.44	1.89
	4	0	27.10	38.33	17.90	7.23	4.77	2.78	1.89
	5	0	31.57	35.73	16.79	6.90	4.57	2.56	1.89

*Note*. — means the machine is breaking down.

**Table 2:** Interaction of sieve sizes and feeding rates for grinding Yang-Na leaves with a pin mill.

Factors	Fineness modulus	Production capacity	SEC	Average particle size	Crude extract yield
Sieve sizes	^*∗∗∗*^	^*∗∗∗*^	^*∗∗∗*^	^*∗∗∗*^	^*∗∗∗*^
Feeding rates	^*∗∗∗*^	^*∗∗∗*^	^*∗∗∗*^	^*∗∗∗*^	^*∗∗∗*^
Sieve sizes × feeding rates	^*∗∗∗*^	^*∗∗∗*^	^*∗∗∗*^	^*∗∗∗*^	^*∗∗∗*^

*Note*. ^*∗∗∗*^*P* ≤ 0.01.

## Data Availability

The data that support the findings of this study are available from the corresponding author upon reasonable request.
